# Learning Breech Birth in an Upright Position Is Influenced by Preexisting Experience—A FRABAT Prospective Cohort Study

**DOI:** 10.3390/jcm10102117

**Published:** 2021-05-14

**Authors:** Lukas Jennewein, Dörthe Brüggmann, Kyra Fischer, Florian J. Raimann, Hemma Roswitha Pfeifenberger, Lena Agel, Nadja Zander, Christine Eichbaum, Frank Louwen

**Affiliations:** 1 Department of Gynecology and Obstetrics, School of Medicine, Goethe-University, Theodor-Stern-Kai 7, 60590 Frankfurt, Germany; Doerthe.Brueggmann@kgu.de (D.B.); Kyra.Fischer@kgu.de (K.F.); HemmaRoswitha.Pfeifenberger@kgu.de (H.R.P.); Christine.Eichbaum@kgu.de (C.E.); louwen@em.uni-frankfurt.de (F.L.); 2 Department of Anaesthesiology, Intensive Care Medicine and Pain Therapy, University Hospital Frankfurt, Goethe University, 60590 Frankfurt, Germany; Florian.Raimann@kgu.de; 3 Carl Remigus Medical School, Limburger Straße 2, 65510 Idstein, Germany; lena.agel@carl-remigius.de (L.A.); Nadja.Zander@web.de (N.Z.)

**Keywords:** vaginal breech, delivery mode, clinical teaching

## Abstract

Background: Vaginal breech delivery is becoming an extinct art although national guidelines underline its safety and vaginal breech delivery in an upright position has been shown to be a safe birth mode option. In order to spread clinical knowledge and be able to implement vaginal breech delivery into obstetricians’ daily practice, we need to gather knowledge from facilities who teach specialized obstetrical management. Methods: We performed a prospective cohort study on 140 vaginal deliveries out of breech presentation solely-managed by seven newly-trained physicians and compared fetal outcome as well as rates of manual assistance in respect to preexisting experience. Results: Fetal morbidity rate measured with a modified PREMODA score was not significantly different in three sub-cohorts sorted by preexisting expertise levels of managing obstetricians (experience groups EG, EG0: 2, 5%; EG1: 3, 7.5%; EG2: 1, 1.7%; *p* = 0.357). Manual assistance rate was significantly higher in EG1 (low experience level in breech delivery and only in dorsal position) compared to EG0 and EG2 (EG1 28, 70%; EG0: 14, 25%; EG2: 21, 35%; *p* = 0.0008). Conclusions: Our study shows that vaginal breech delivery with newly-trained obstetricians is a safe option whether or not they have advanced preexisting expertise in breech delivery. These data should encourage implementing vaginal breech delivery in clinical routine.

## 1. Introduction

Only a few obstetrical institutions offer clinical care for vaginal breech delivery. One obvious reason is that only 4–5% of all pregnant women carry their baby in breech presentation at term [1], and low prevalence triggers centralization of cases in centers with expertise in this particular field. Due to this limited clinical exposure, conflicting evidence regarding perinatal morbidity and fear of medicolegal consequences, the comfort level of many obstetricians to supervise vaginal breech births in a hospital setting is low. Hence, cesarean section rates in breech pregnancies at term are estimated to be above 80% [2]. This seems not justified since the adverse long-term outcome (neurodevelopmental delay after two years) was reported not to be different between elective cesarean sections and vaginal births with an RR of 1.09 [3], underlying the safety of vaginal breech deliveries.

Not every obstetrical department should feel obliged to offer care for vaginal breech deliveries on a routine basis. Nevertheless, each and every obstetrician should be equipped to handle a delivery of a breech fetus. Undetected breech presentations can surprise the laborist (e.g., during precipitate deliveries) anytime and everywhere; if these situations are handled poorly, the consequences are disastrous. Various national breech guidelines exist to guide counseling and clinical management, but they differ in their recommendations [4,5,6]. However, vaginal breech delivery can be a considerable option for a carefully selected patient clientele. Numerous papers were published in the past few years that clearly underline the safety of vaginal breech delivery—particularly in an upright birth position—regarding neonatal and maternal outcomes and these refute guideline-based restrictions and concerns. In this regard, nulliparity, high birth weight, overdue pregnancy, birth induction and previous cesarean section do not seem to affect neonatal or maternal morbidity substantially [7,8,9,10,11,12]. More importantly, slightly elevated rates in short-term morbidity did not translate into long-term morbidity in vaginally intended breech deliveries [13]. In light of these findings, cesarean section for breech birth should not be the general recommendation and ultimate choice for patients expecting a breech fetus. The “rethinking of breech delivery” is an important factor in the global battle to decrease cesarean section numbers which are constantly rising and paralleled by increasing subsequent morbidity for mother and child. Here, not only peri-operative risks but also possible complications in following pregnancies have to be considered [14,15,16].

Since clinical expertise and teaching competency for vaginal breech delivery are generally dwindling, it is of high importance to promote the training of obstetricians in this clinical skill around the world. The quality and frequency of teaching how to deliver a baby out of breech presentation is improvable [17,18]. To amplify teaching of the management of vaginal breech delivery in an upright position, it is necessary to study delivery outcomes and learning curves of newly trained obstetricians and to take a closer look on the preexisting knowledge and training. We performed a prospective cohort study on the first 20 deliveries solely-managed by seven newly-trained obstetricians in our center (*N* = 140). Our objective was to analyze a learning curve within the first 20 deliveries and to look at how preexisting knowledge influences delivery management. Herein, we analyze birth outcome in respect to the obstetrician’s preexisting expertise. This study gives valuable input to obstetrical departments who are implementing and teaching the vaginal breech delivery in an upright position.

## 2. Materials and Methods

### 2.1. Patient Cohort and Patient Selection

From January 2004 until December 2019, we performed a prospective case-controlled study on vaginal deliveries of term singletons in breech presentation (>37 0/7 weeks of pregnancy) at the Goethe University Hospital in Frankfurt. The university hospital’s ethics committee gave consent (420/11). All patients were treated within standard clinical care. Thus, the ethics committee waived a particular patient’s consent; 140 solely-managed deliveries of seven different newly-trained laborists were included in this study.

### 2.2. Breech Birth Training

The obstetricians who supervised vaginal breech delivery in our center were all certified for “Maternal Fetal Medicine” by the German Medical Board. They attended at least 10 vaginal breech deliveries in our center and then managed 10 deliveries under supervision of a senior teacher. After this training period, obstetricians were included in this study. Some of these physicians collected experience in the care of vaginal breech deliveries in their former workplace. We generated 3 groups in respect to the obstetricians’ preexisting experience (none = 0; low, meaning few experience with breech deliveries only in dorsal position = 1; advanced, meaning already experience with breech delivery in upright birth position = 2).

### 2.3. Data Collection

For data collection, the state database ’Perinatalerhebung Hessen’ was used. Data was completed using the hospital’s patient management system. All data was gathered after discharge of patients.

The counseling process, the FRABAT cohort, the modified PREMODA morbidity score as well as patient selection and procedures at our center have been previously described [7,8,9,10,11].

The preferred maternal position during stage two of delivery in all vaginal deliveries was the upright position. In rare cases, the managing obstetrician changed birth position individually in order to perform classical breech delivery maneuvers. In order to gain the best possible statistical quality in a single center, we included as many cases as possible in the respective time frame—keeping equal sample sizes per managing obstetrician. A post hoc power analysis revealed a Power of 97.3% with our sample size (primary endpoint: manual assistance rate, alpha = 0.05).

### 2.4. Statistical Analyses

Groups of variables were tested with normal distribution applied with Kolmogorov—Smirnov testing. Group differences were tested using Pearson’s χ^2^ test. Continuous variables were compared using *t*-testing. Statistical analyses were conducted using JMP 14.0 software (SAS Institute, Cary, NC, USA). A *p*-value of below 0.05 was considered as statistically significant.

## 3. Results

Our cohort consisted of 140 vaginal breech deliveries, managed by 7 different obstetricians. There were 77 (55%) spontaneous vaginal deliveries and 63 (45%) manually assisted deliveries. In total, 28 patients (20%) were turned on their back in order to perform classical breech birth maneuvers. This was by choice of the attending obstetrician (Table 1).

The seven managing obstetricians had a different preexisting experience level concerning breech deliveries. Two had no experience (0), two had only little experience and only with deliveries with mothers on their backs (1) and three obstetricians had already advanced experience in managing breech birth, including breech delivery in an upright position (2).

Parity, BMI, fetal birth weight and peridural anesthesia during birth and perineal injury rates did not significantly differ between deliveries managed by the seven obstetricians (Table 2). Delivery mode, however, did differ significantly between obstetricians. Obstetricians 1, 2, 6 and 7 had significantly lower manual assistance rates (4 (20%), 7 (35%), 7 (35%), 7 (35%)) than the other obstetricians (Table 2).

Parity and fetal birth weight were not significantly different between experience group 0 (EG0), experience group 1 (EG1) and experience group 2 (EG2). Fetal outcome parameters were also not significantly different: A stay on the neonatal intensive care unit (NICU) of more than four days (EG0: 3, 7.5%; EG1: 2, 5%; EG2: 4, 7.5%, *p* = 0.897), 5 min APGAR score below 4 (EG0: 1, 2.5%; EG1: 0, 0%; EG2: 0, 0%; *p* = 0.284), fetal intubation period of more than 24 h (EG0: 2, 5%; EG1: 0, 0%; EG2: 1, 1.7%; *p* = 0.628). We calculated a modified PREMODA score to simplify newborn morbidity. A case counts as a “morbidity case” when one or more of the following criterions are met: 5 min APGAR score below 4, intubation for more than 24 h, stay on the NICU for more than 4 days. The case was not counted as “birth mode associated morbidity” if an infection or congenital disease were the cause of the newborn’s morbidity, resulting in the modified PREMODA score possibly related to birth mode. Modified (Mod.) PREMODA score values were not significantly different between groups in this analysis (EG0: 2, 5%; EG1: 3, 7.5%; EG2: 1, 1.7%; *p* = 0.357) (Table 3).

Rates of manual assistance performed by the obstetrician were significantly higher in EG1 (28, 70%) compared to both other groups (EG0: 14, 25%; EG2: 21, 35%) with a *p*- value of 0.0008. Rates of maneuvers to help with arm or head delivery parallel this finding (Table 3). Rates of mothers, who delivered on their backs in order to perform classical breech birth maneuvers, were significantly higher in EG1 (19, 47.5%) compared to both other groups (EG0: 1, 2.5%; EG2: 9, 15%; *p* > 0.0001). Deliveries in EG1 were significantly more often performed with peridural anesthesia (EG1: 27, 67.5%; EG0: 16, 40%; EG2: 32, 53.3%; *p* = 0.048). Rates of perineal injury showed a non-significant tendency towards a higher rate of perineal injuries in EG1 (EG1: 25, 62.5%; EG0: 15, 37.5%; 25, 41.7%, *p* = 0.05).

To display a learning curve, the first 10 deliveries were compared to the following 10. Deliveries managed by all obstetricians were analyzed this way. Parity and fetal birth weight were not significantly different between the two groups. Fetal morbidity parameters—including the modified PREMODA score related to birth mode—were not different comparing deliveries 1–10 versus deliveries 11–20 (1–10: 3, 4.3%; 11–20: 3, 4–3%; *p* = 1.0; Table 4). The rate of general manual assistance (assistance for head and/or arm delivery) performed during birth was significantly higher in deliveries 11–20 (40, 57.1%) compared to deliveries 1–10 (23, 32.9%, *p* = 0.004). Help to deliver the arms was performed to the same extent in both groups (1–10: 18, 25.7%; 11–20: 26, 37.1%; *p* = 0.145), while maneuvers to accelerate head delivery were performed more often in deliveries 11–20 (33, 47.1%) in comparison to deliveries 1–10 (21, 30%; *p* = 0.037; Table 4). The rate of mothers turned into dorsal position was not significantly different between the groups (1–10: 11, 15.71%; 11–20: 18, 25.7%; *p* = 0.144). Peridural anesthesia during birth was applied in 31 (44.3%) cases in deliveries 1–10 and in 44 (62.9%) cases in deliveries 11–20 (*p* = 0.028). Perineal injuries were equally distributed in this analysis (1–10: 35, 50%; 11–20: 30, 42.9%; Table 4).

To determine whether preexisting knowledge influences a learning behavior within the first 20 deliveries, we analyzed deliveries 1–10 and 11–20 within the three experience groups.

Fetal morbidity was not different in between the experience groups when deliveries 1–10 were compared to deliveries 11–20 (Table 5A–C). There was a significant increase in the rate of manual assistance in EG0 (1–10: 3, 15%; 11–20: 11, 55%; *p* = 0.008) and EG1 (1–10: 11, 55%; 11–20: 17, 85%; *p* = 0.0384) but not in EG2 (1–10: 9, 30%; 11–20: 12, 35%; *p* = 0.417). In the group with the lowest preexisting experience, help of delivery of the head was performed significantly more often in deliveries 11–20 (10, 50%) compared to deliveries 1–10 (1, 5%, *p* = 0.0014) (Table 5). Rates of manual assistance of the head delivery were not significantly different in both other experience groups. The decision to turn the birthing mother on her back into dorsal position did not occur significantly more or less often when deliveries 1–10 and 11–20 were compared in all three experience groups (Table 5). Additionally, perineal injury rates did not differ between delivery cohorts within the three different experience groups (Table 5).

## 4. Discussion

Learning about the impact of preexisting experience on the learning curve in the management of vaginal breech delivery is important in order to facilitate the implementation of a secure birth mode and reduce alarmingly high cesarean section rates worldwide. This study is the first to report on delivery management outcome in respect to the obstetrician’s experience in vaginal breech deliveries. The aim was to show the safety of vaginal breech birth managed by newly-trained obstetricians and to analyze how preexisting experience may influence birth management.

Age, BMI and parity, which generally influence birth outcome [7,9], were equally distributed within the managed deliveries of seven different obstetricians (Table 2). The rates of manual assistance during birth were significantly depending on the managing obstetrician, showing a varying tendency to intervene (Table 2). Manual intervention was significantly more often applied when obstetricians had low experience (EG1) compared to obstetricians with no (EG0) or advanced experience (EG2) (Table 3). Obstetricians with a low experience level learned to assist manually in each and every delivery because they were predominantly taught vaginal delivery in dorsal position. This position requires manipulation through the birth attendant in order to deliver the baby. This habit seems to translate into the same “hands-on” management of a breech birth with a mother on all fours—even though maneuvers are often not necessary when mothers give birth in an upright position. Additionally, obstetricians with low experience (EG1) significantly more often turned mothers on their backs in order to perform manual assistance (Table 3). The obvious explanation is that they learned to manage vaginal breech births only in dorsal position and felt more comfortable this way. Additionally, it is harder to adapt when the obstetrician is neither influenced by knowledge (EG0), nor has already a lot of experience (EG2).

We compared the first ten deliveries to the following ten in order to observe a learning curve. Again, parity, BMI and fetal birth weight were not different in both groups, underlining comparability (Table 4). Fetal morbidity, measured with a modified PREMODA Score adapted from Goffinet et al. [13,19] and used in previous publications [7,8,9,10,11] did not show a significant difference. Since obstetricians have had training before supervising vaginal breech birth on their own, a difference in fetal outcome in this analysis would have been surprising and of great concern. Additionally, after dividing these two cohorts based on preexisting experience, no difference in fetal morbidity rates were observed, displaying a teaching success in all experience groups and underlining vaginal breech safety. Manual assistance rate was higher in deliveries 11–20 compared to the first ten (Table 4). Remarkably, rates of maneuvers assisting arm delivery did not occur more often, while head delivery maneuvers such as the Frank Nudge maneuver were performed significantly more often in deliveries 11–20 (Table 4). The sub-cohort analysis of deliveries 11–20 and 1–10 in EG02 revealed that the increase of manual assistance during breech delivery is apparent in EG0 and EG1 but not in EG2 (Table 5). Hence, obstetricians with a lower experience level seem to be less reluctant to perform maneuvers accelerating head delivery.

Cesarean section rate in pregnancies with breech presentation is reported to be above 80% [2]. In our center, 1/3 of women presenting with breech presentation decided to have an elective cesarean section after thorough counseling for all delivery options [11]. The cesarean section rate after onset of labor and vaginal birth intent is 25% and generally due to non-reassuring fetal status or arrest in labor. As shown in our study, teaching the clinical management of vaginal breech births in a structured and standardized approach does not take years of time. Attending 10 vaginal breech births managed by an experienced practitioner and managing 10 vaginal births under supervision lays a fundament to safely and successfully managing vaginal breech birth of patients who were appropriately selected and counseled. Hence, we want to underline the possibility to successfully implement a breech birth teaching program in other obstetrical departments, which would lead to a viable reduction of cesarean section rates and contribute to fewer cesarean section-associated complications [20]. The desire of pregnant patients to be counseled thoroughly with up-to-date evidence [21,22] regarding the different delivery modes of a breech baby and to select a delivery mode tailored to their individual preferences is increasing. In order to answer this demand for vaginal birth options by our patients, it is mandatory to regain experience in vaginal breech delivery.

Limitations of this study include the fact that this is a single center study. Teaching methods and delivery management might not be translatable to other clinical departments. The sample size of the sub-cohort analyses is quite low. In order to improve reliability, we need multiple center studies with higher delivery numbers and standardized teaching protocols. Deliveries themselves differ individually every time, which makes an inter-operator variability analysis difficult. Thus, results should be interpreted with caution. The personal working experience period was not taken into account. It is possible that solely the period of working as an obstetrician might impact birth management, independently from experience in breech delivery. Breech delivery is a birth managed by physicians in our center. Midwifes can also have great impact on delivery outcome—this aspect has not been elucidated in this study.

Our data clearly shows that vaginal breech delivery with newly trained obstetricians is a safe option whether or not they have advanced experience in breech delivery since fetal morbidity rates are not different. The standardized hands-on teaching of vaginal breech delivery in our center seems to prepare various obstetricians to perform consistently in managing vaginal breech deliveries. We see that obstetricians with low experience in vaginal breech delivery, who learned to deliver in dorsal position with maneuvers, tend to assist manually more often. These data should encourage other clinics to implement a teaching module for their faculty and residents to gain confidence and comfort in handling a vaginal breech delivery safely, e.g., in a precipitate birth or with carefully selected patients. Moreover, this study should encourage permanently implementing the skill of vaginal breech birth management in our clinical routine with the long-term aim of reducing cesarean section numbers. Teaching should be adapted in respect to preexisting experience levels, with an emphasis on reducing the intervention rate in the upright birth position.

## Figures and Tables

**Table 1:** Delivery variables of the whole study cohort.

Variable	Cohort, *N* = 140
Age (mean, st.dev.)	32.2 (4.1)
BMI (mean, st.dev.)	22.6 (3.8)
Duration of pregnancy in weeks	40 (1)
Parity (n, %)	
1	72 (51.4)
2	48 (34.3)
>2	20 (14.3)
Fetal birth weight (gram; mean, st.dev.)	3340 (±412)
PDA	75 (54%)
Perineal injury	65 (46%)
Delivery mode	
Spontaneous vaginal birth	77 (55%)
Manually assisted birth	63 (45%)
Manually assisted birth in dorsal position	28 (20%)

**Table 2:** Delivery variables of cases managed by seven different obstetricians.

Variable	1 *N* = 20	2 *N* = 20	3 *N* = 20	4 *N* = 20	5 *N* = 20	6 *N* = 20	7 *N* = 20	*p*-Value
Experience	2	1	2	2	1	0	0	
Parity								0.195
1	12 (60%)	14 (70%)	11 (55%)	10 (50%)	10 (50%)	10 (50%)	5 (24%)	
2	5 (25%)	4 (20%)	4 (20%)	8 (40%)	9 (45%)	6 (30%)	12 (60%)	
>2	3 (15%)	2 (10%)	5 (25%)	2 (10%)	1 (5%)	4 (20%)	3 (15%)	
BMI (mean, st.dev.)	23.6 (±3.2)	22.1 (±1.8)	21.6 (±3.1)	22.1 (±4.6)	24.9 (±6.2)	22.1 (±2.9)	22.0 (±2.4)	0.06
Fetal birth weight (gram; mean, st.dev.)	3333 (±363)	3382 (±435)	3274 (402)	3263 (±351)	3471 (±385)	3283 (±427)	3379 (±524)	0.707
PDA	10 (50%)	11 (55%)	12 (60%)	10 (50%)	16 (80%)	7 (35%)	9 (45%)	0.146
Perineal injury	10 (50%)	11 (55%)	6 (30%)	9 (45%)	14 (70%)	8 (40%)	7 (35%)	0.189
Delivery mode								0.006
Spontaneous vaginal birth	16 (80%)	7 (35%)	13 (65%)	10 (50%)	5 (35%)	13 (65%)	13 (65%)	
Manually assisted birth	4 (20%)	13 (65%)	7 (35%)	10 (50%)	15 (65%)	7 (35%)	7 (35%)	

**Table 3:** Comparison of deliveries between experience groups.

Variable	EG0 (*N* = 40)	EG1 (*N* = 40*)*	EG2 (*N* = 60)	*p*-Value
Parity (n, %)				0.196
1	15 (37.5%)	24 (60%)	33 (55%)	
2	18 (45%)	13 (32.5%)	17 (28.3%)	
>2	7 (17.5%)	3 (7.5%)	10 (7.5%)	
Fetal birth weight (gramm; mean, st.dev.)	3331 (475)	3426 (408)	3290 (368)	0.287
NICU > 4 days	3 (7.5%)	2 (5%)	4 (6.7%)	0.897
5 min APGAR < 4	1 (2.5%)	0 (0.0%)	0 (0.0%)	0.284
Intubation > 24 h	1 (2.5%)	0 (0%)	1 (1.7%)	0.628
Fetal infection	2 (5%)	3 (7.5%)	3 (5%)	0.847
Mod. PREMODA Score	4 (10%)	5 (12.5%)	4 (6.7%)	0.606
Mod. PREMODA Score possibly related to birth mode	2 (5%)	3 (7.5%)	1 (1.7%)	0.357
Manual assistance	14 (35%)	28 (70%)	21 (35%)	0.0008
Help with delivery of arms	7 (17.5%)	24 (60%)	13 (21.7%)	<0.0001
Help with delivery of the head	11 (27.5%)	24 (60%)	19 (31.7%)	0.004
Frank Nudge	9 (22.5%)	2 (5%)	7 (11.7%)	0.061
Dorsal position	1 (2.5%)	19 (47.5%)	9 (15%)	<0.0001
PDA	16 (40%)	27 (67.5%)	32 (53.3%)	0.048
Perineal injury	15 (37.5%)	25 (62.5%)	25 (41.7%)	0.050

**Table 4:** Comparison of deliveries 1–10 and 11–20.

Variable	Deliveries 1–10 (*N* = 70)	Deliveries 11–20 (*N* = 70)	*p*-Value
Parity (n, %)			0.308
1	34 (48.6%)	38 (54.3)	
2	29 (40%)	20 (28.6%)	
>2	8 (11.4%)	12 (17.4%)	
Fetal birth weight (gram; mean, st.dev.)	3345 (370)	3336 (454)	0.796
NICU > 4 days	4 (5.7%)	5 (7.1%)	0.730
5 min APGAR < 4	0 (0%)	1 (1.4%)	0.316
Intubation >24 h	1 (1.43%)	1 (1.43%)	1.000
Fetal infection	3 (4.3%)	5 (7.1%)	0.467
Mod. PREMODA Score	6 (8.6%)	7 (10%)	0.771
Mod. PREMODA Score possibly related to birth mode	3 (4.3%)	3 (4.3%)	1.000
Manual assistance	23 (32.9%)	40 (57.1%)	0.004
Help with delivery of arms	18 (25.7%)	26 (37.1%)	0.145
Help with delivery of the head	21 (30%)	33 (47.1%)	0.037
Frank Nudge	4 (5.7%)	14 (20%)	0.012
Dorsal position	11 (15.71%)	18 (25.7%)	0.144
PDA	31 (44.3%)	44 (62.9%)	0.028
Perineal injury	35 (50%)	30 (42.9%)	0.718

**Table 5:** Comparison of deliveries 1–10 and 11–20 in experience sub-cohorts.

Variable	Deliveries 1–10	Deliveries 11–20	*p*-Value
**(A) Experience = 0**	***N* = 20**		***N* = 20**
Mod. PREMODA poss. related to BM	0 (0%)	2 (10%)	0.147
Manual assistance	3 (15%)	11 (55%)	0.0080
Help with delivery of arms	2 (10%)	5 (25%)	0.212
Help with delivery of the head	1 (5%)	10 (50%)	0.0014
Frank Nudge	0 (0%)	9 (45%)	0.0007
Dorsal position	0 (0%)	1 (5%)	0.311
PDA	7 (35%)	9 (45%)	0.519
Perineal injury	8 (40%)	7 (35%)	0.744
**(B) Experience = 1**	***N* = 20**		***N* = 20**
Mod. PREMODA poss. related to BM	2 (10%)	1 (5%)	0.548
Manual assistance	11 (55%)	17 (85%)	0.0384
Help with delivery of arms	10 (50%)	14 (70%)	0.197
Help with delivery of the head	11 (55%)	13 (65%)	0.519
Frank Nudge	2 (10%)	0 (0%)	0.147
Dorsal position	8 (40%)	11 (55%)	0.342
PDA	14 (70%)	13 (65%)	0.736
Perineal injury	13 (65%)	12 (60%)	0.774
**(C) Experience = 2**	***N* = 20**		***N* = 20**
Mod. PREMODA poss. related to BM	1 (0%)	0 (0%)	0.313
Manual assistance	9 (30%)	12 (40%)	0.417
Help with delivery of arms	6 (20%)	7 (23.3%)	0.754
Help with delivery of the head	9 (30%)	10 (33.3%)	0.781
Frank Nudge	2 (6.7%)	5 (16.7%)	0.228
Dorsal position	3 (10%)	6 (20%)	0.278
PDA	10 (33.3%)	22 (73.3%)	0.002
Perineal injury	14 (46.7%)	11 (36.7%)	0.432

## Data Availability

All data will be made available on DRYAD platform after acceptance.
